# Successful Early Therapy With Sirolimus for an Infant With Primary Intestinal Lymphangiectasia

**DOI:** 10.1155/crpe/7717714

**Published:** 2026-09-15

**Authors:** Shungo Yamamoto, Yoshihiko Sugino, Takahiro Yamashita, Kei Honda, Yugo Takaki

**Affiliations:** ^1^ Department of Pediatrics, Japanese Red Cross Kumamoto Hospital, Kumamoto, Japan, jrc.or.jp; ^2^ Department of Pediatric Gastroenterology and Hepatology, Japanese Red Cross Kumamoto Hospital, Kumamoto, Japan, jrc.or.jp

**Keywords:** infant, primary intestinal lymphangiectasia, protein-losing enteropathy, sirolimus

## Abstract

**Introduction:**

Identifying the cause of protein‐losing enteropathy (PLE) is crucial for appropriate treatment. Primary intestinal lymphangiectasia (PIL) is a cause of PLE, and low‐strict fat diet with medium‐chain triglyceride (MCT) supplementation may be insufficient. Sirolimus has shown efficacy in refractory PIL; however, its use in infants remains uncommon, and the optimal timing of initiation has not been established.

**Case Presentation:**

A previously healthy 7‐month‐old Japanese male infant presented with convulsions, generalized edema, and diarrhea. Laboratory findings revealed severe hypoproteinemia (serum albumin: 1.3 g/dL), lymphopenia (absolute lymphocyte count: 822/μL), and electrolyte abnormalities. Scintigraphy using ^99m^Tc‐labeled human serum albumin showed tracer exudate in the small intestine, leading to the diagnosis of PLE. Upper endoscopy revealed snowflake‐like white spots in the duodenum, and pathological examination confirmed lymphatic dilation, leading to a PIL diagnosis. Despite treatment with a low‐strict fat diet with MCT supplementation and corticosteroids, hypoproteinemia persisted. Lymphatic scintigraphy and magnetic resonance lymphangiography confirmed ongoing protein leakage. Sirolimus was initiated at 0.5 mg/day on Day 23. Subsequently, serum albumin began to increase within 1–2 weeks and ultimately reached 4.1 g/dL, and absolute lymphocyte count, 1917/μL immediately before sirolimus initiation, increased to 3843/μL. Lymphatic scintigraphy on Day 74 of sirolimus treatment showed complete resolution of protein leakage. Sirolimus was continued for 121 days and discontinued thereafter. At 17 months of follow‐up, no recurrence was observed, with sustained normalization of serum albumin to 4.0 g/dL and absolute lymphocyte count to 4739/μL.

**Discussion:**

This case suggests one of the earliest reported responses to sirolimus in the treatment of PIL, with improvement observed within a few weeks of initiation. Early introduction of sirolimus in patients with persistent protein leakage resistant to low‐strict fat diet with MCT supplementation may be associated with rapid improvement in PIL, though this hypothesis warrants further case accumulation.

## 1. Introduction

Protein‐losing enteropathy (PLE) is a condition in which excessive protein leakage from the gastrointestinal tract leads to nonselective hypoproteinemia, and identifying its underlying cause is clinically important [1]. Primary intestinal lymphangiectasia (PIL) is one such cause, a sporadic congenital disorder characterized by pathological dilation of intestinal lymphatic vessels. Reported cases worldwide remain scarce, and although the actual prevalence has yet to be established, the condition is most frequently identified during early childhood, particularly within the first 3 years of life, with a slight male predominance [2, 3]. A nationwide French survey identified only 34 cases over 12 years, underscoring the rarity of this condition and the practical impossibility of conducting prospective trials [2, 4].

The fundamental pathological process involves leakage of chyle into the intestinal lumen, leading to PLE and consequent hypoalbuminemia, hypogammaglobulinemia, lymphopenia, and edema [2]. At the molecular level, aberrations in genes governing lymphatic endothelial differentiation and maturation—including vascular endothelial growth factor receptor–3 (VEGFR3), prospero‐related homeobox‐transcriptional factor–1 (PROX1), forkhead box transcription factor C2 (FOXC2), and sex‐determining region Y‐box transcription factor–18 (SOX18)—have been implicated, along with dysregulation of the hepatocyte growth factor/mesenchymal–epithelial transition factor (HGF/MET) signaling pathway; however, the precise pathophysiological mechanisms underlying PIL remain incompletely understood [3]. A recent multicenter cohort study demonstrated that the presence of lymphedema or a pathogenic genetic variant was significantly associated with only a partial response to dietary therapy, highlighting the growing need for molecularly informed, individualized treatment strategies [2].

No universally accepted treatment guidelines for PIL have been established, and management is primarily guided by institutional experience and available literature. The cornerstone of first‐line therapy is a diet restricting long‐chain triglycerides supplemented with medium‐chain triglycerides (MCTs), with a response rate of 81% reported in a French nationwide cohort (*n* = 34) [2]. For localized disease, surgical resection or lymphatic embolization has achieved complete remission in selected cases [4]. When dietary therapy proves insufficient in patients with extensive disease, octreotide or sirolimus is considered. Octreotide, a somatostatin analog, was associated with resolution of diarrhea and reduced albumin infusion requirements in all patients in one pediatric series, with normalization of serum albumin achieved in 50% [5]; however, its efficacy is limited in cases with extraintestinal lymphatic involvement, and symptomatic relapse following discontinuation has been documented [4]. Sirolimus exerts antilymphangiogenic effects through inhibition of mammalian target of rapamycin (mTOR) signaling in lymphatic endothelial cells, and clinical improvement within one to several months of initiation has been reported in extensive‐type PIL; nevertheless, no consensus exists regarding the optimal timing of introduction or duration of sirolimus therapy, and experience with its use in infants remains explicitly scarce [3, 4].

Given the limited experience with sirolimus in infants with PIL and the uncertain efficacy of early intervention, we report a case in which sirolimus was introduced at an early stage in an infant with PIL refractory to a low‐strict fat diet with MCT supplementation, resulting in clinical and biochemical improvement within 1–2 weeks of treatment initiation.

## 2. Case Presentation

**FIGURE 1:**
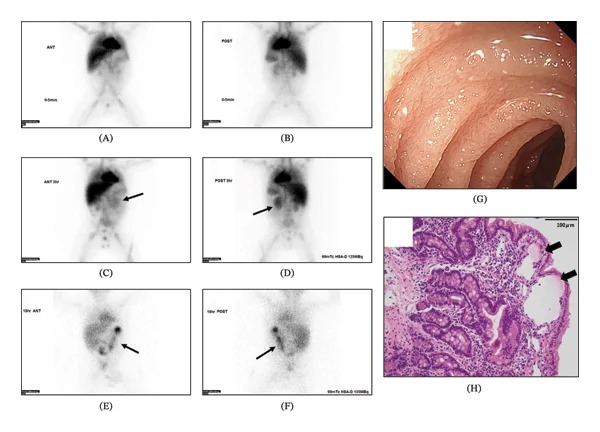
Protein‐losing scintigraphy on Day 5 in anterior (A, C, and E) and posterior views (B, D, and F). Immediately after intravenously injecting ^99m^Tc‐labeled human serum albumin (HSA), no accumulation of nuclides is observed (A, B). After 3 h, albumin leakage into the small intestine (arrow) is evident (C, D). After 19 h, leaked albumin is shown to migrate from the splenic flexure to the sigmoid colon (arrow) (E, F). Upper endoscopy on Day 6 reveals snowflake‐like white spots on the duodenum (G). Pathological examination reveals irregularly dilated lymph vessels in the mucosal lamina propria (arrow) (H).

A previously healthy 7‐month‐old Japanese boy with no significant medical or perinatal history presented to a local hospital with afebrile convulsions. Seizures were initially controlled with midazolam administration. On admission, anthropometric measurements were within normal ranges for age (height: −0.07 SD; weight: +0.61 SD). Laboratory examination at the referring hospital revealed hypocalcemia and hypoproteinemia. He was then transferred to our hospital by ambulance for further evaluation and management. Family history was notable only for allergic rhinitis in both parents, with no family history of lymphatic disorders. Laboratory parameters were as follows: white blood cell count, 4213/μL (lymphocytes: 19.5%; absolute lymphocyte count: 822/μL); total protein, 2.8 g/dL; albumin, 1.3 g/dL; immunoglobulin G (IgG), 14 mg/dL (normal: 300–960 mg/dL); IgA, 13 mg/dL (normal: 9–55 mg/dL); IgM, 13 mg/dL (normal: 51–188 mg/dL); calcium, 5.3 mg/dL; and magnesium, 1.0 mg/dL. Urinalysis showed no proteinuria. Ultrasonography revealed no pleural or pericardial effusions or ascites. Hypocalcemia was corrected with intravenous calcium, resulting in cessation of convulsions. Although alpha‐1‐antitrypsin was not appropriately assessed because of difficulties in collecting diarrheal stool, protein‐losing scintigraphy (intravenous injection of ^99m^Tc‐labeled human serum albumin [HSA]) on Day 5 showed tracer exudation in the small intestine at 3 h, indicating PLE (Figures 1A–F). Upper endoscopy on Day 6 revealed snowflake‐like white spots on the duodenum (Figure 1G); pathological examination revealed chronic inflammation and irregularly dilated lymph vessels in the mucosal lamina propria (Figure 1H). Secondary intestinal lymphangiectasia was excluded. PIL was definitively diagnosed based on the characteristic clinical presentation, imaging findings (protein‐losing scintigraphy and magnetic resonance lymphangiography [MRL]), histopathological evidence of lymphatic dilation, and the absence of secondary causes. The patient was administered nutritional therapy restricting long‐chain fatty acids, including fasting from Day 5, followed by initiation of MCT formula on Day 10. Specifically, he received Meiji Essential Fatty Acid–Fortified MCT Formula at 14% concentration, with a daily intake of 900–1500 mL. Corticosteroids were administered empirically to reduce intestinal permeability and possible inflammatory components: intravenous prednisolone (PSL) at 10 mg daily was started on Day 6 and switched to oral PSL 10 mg on Day 13.

**FIGURE 2:**
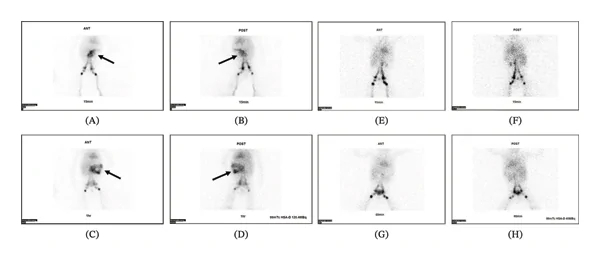
Lymphatic scintigraphy is performed on Day 17, before induction of sirolimus (A–D) and on Day 96 (Day 74 of sirolimus administration) (E–H). Lymphatic scintigraphy on Day 17 shows persistent leakage from the intestinal tract into the abdominal cavity (arrow) (A–D). Lymphatic scintigraphy on Day 96 shows no leakage (E–H). Anterior (A) and posterior (B) views at 15 min and anterior (C) and posterior (D) views at 1 h on day 17 are shown. Anterior (E) and posterior (F) views at 15 min and anterior (G) and posterior (H) views at 1 h on Day 96 are shown.

**FIGURE 3:**
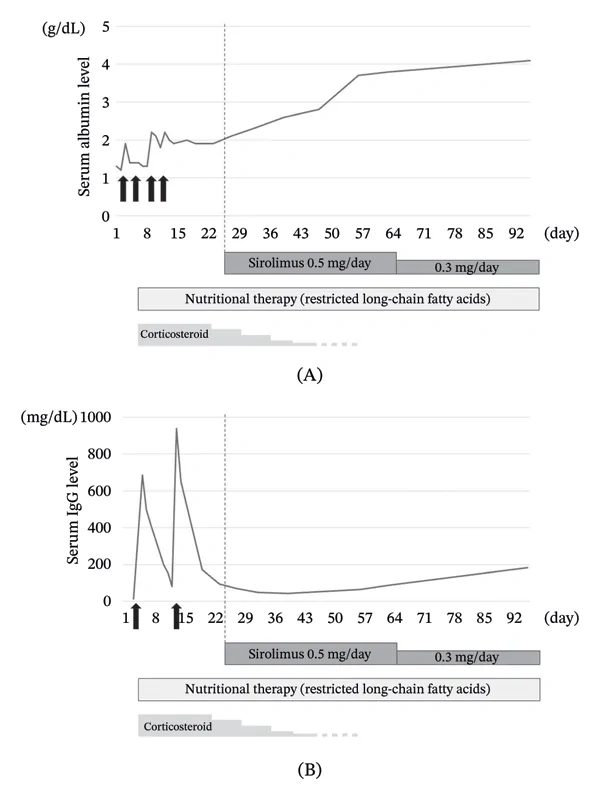
Serum albumin levels during sirolimus therapy (A). The arrow indicates intravenous albumin infusion (10 g). Serum IgG levels during sirolimus therapy (B). The arrow indicates intravenous immunoglobulin infusion (5.0 g). The horizontal axis indicates the time from the start of sirolimus administration.

These treatments improved electrolyte abnormalities; however, hypoproteinemia persisted. Noncontrast MRL on Day 16 and lymphatic scintigraphy (subcutaneous injection of ^99m^Tc‐labeled HSA) on Day 17 revealed persistent leakage from the intestinal tract into the abdominal cavity. No leakage was observed at other sites (Figures 2A–D). Given the sustained hypoalbuminemia and ongoing lymphatic leakage, the condition was deemed refractory to a low‐strict fat diet with MCT supplementation. PSL was gradually tapered and discontinued (7.5 mg/day on Day 21, 5 mg/day on Day 28, 2.5 mg/day on Day 35, 1 mg/day on Day 40, and 1 mg on alternate days from Day 47 for 6 days). Subsequently, sirolimus was administered at 0.5 mg/day (1.2 mg/m^2^/day) starting on Day 23, according to the Japanese package insert dosing recommendations for pediatric patients. Prior to initiation, the parents were counseled by both the physician and the pharmacist regarding the risks of long‐term immunosuppression, including potential adverse effects such as interstitial pneumonia, infections, and oral ulceration. Two weeks after initiation, the blood sirolimus level was 7.2 ng/mL, within the target therapeutic range (5–15 ng/mL). Subsequently, the patient’s serum albumin began to increase within 1–2 weeks of sirolimus initiation and ultimately reached 4.1 g/dL; IgG levels rose (Figure 3A, B), and the absolute lymphocyte count, which was 1917/μL prior to sirolimus initiation, increased to 3843/μL during treatment. On Day 52, sirolimus dosage was reduced to 0.3 mg/day due to elevated creatine kinase; however, the causal relationship remains unclear. Creatine kinase levels gradually normalized following dose reduction. Lymphatic scintigraphy on Day 96 (Day 74 of sirolimus) revealed no leakage into the abdominal cavity (Figures 2E–H). Given the prior adverse event and the absence of lymphatic leakage, we planned to discontinue sirolimus if further adverse events occurred. Sirolimus was discontinued on Day 143 following the onset of fever suggestive of an upper respiratory infection. No other adverse events attributable to sirolimus were observed during treatment. At approximately 17 months of follow‐up, the patient remained free from recurrence with no edema or diarrhea. Growth and developmental milestones were age‐appropriate, with height +0.78 SD and weight +0.15 SD. Concurrent laboratory findings revealed sustained normalization of serum albumin to 4.0 g/dL, IgG to 1401 mg/dL, total protein to 7.7 g/dL, and absolute lymphocyte count to 4739/μL after sirolimus discontinuation. Favorable nutritional status was confirmed based on satisfactory growth, typical developmental milestones, and laboratory findings following treatment.

## 3. Discussion

PLE increases intestinal protein loss, leading to nonselective hypoproteinemia [1, 5, 6]. In this patient, PLE was suspected because of nonselective hypoproteinemia, electrolyte and trace element deficiencies, edema, and diarrhea. Although alpha‐1‐antitrypsin clearance is an important diagnostic test for PLE, adequate 3‐day fecal collection could not be obtained despite multiple attempts due to frequent diarrhea. PLE was diagnosed based on radiotracer accumulation in the intestine on ^99m^Tc‐labeled HSA scintigraphy. PLE has many possible causes, including intestinal lymphangiectasia [1, 6].

Intestinal lymphangiectasia is classified into primary and secondary forms. PIL is a congenital lymphatic disorder characterized by dilation and tortuosity of the intestinal lymphatics because of impaired lymphatic drainage [7]. According to the diagnostic framework proposed by Vignes and Bellange [3, 8], PIL is diagnosed based on histopathological confirmation of intestinal lymphangiectasia, clinical and laboratory evidence of PLE, and exclusion of secondary causes, which include cardiac disease, malignancy, and infection [6]. In this case, D‐xylose testing, quantitative fecal fat measurement, and fecal pancreatic elastase–1 assay were not performed due to difficulties with stool collection and a lack of standardization in routine practice, which limited the ability to characterize the mechanisms of malabsorption fully. Nevertheless, a comprehensive evaluation, including echocardiography, abdominal imaging, and pathological examination, revealed no secondary causes, establishing the diagnosis of PIL. Genetic mutations in *CCBE1*, *FAT4*, and *ADAMTS3* have been identified in syndromic forms of intestinal lymphangiectasia, such as Hennekam syndrome [2, 9], while Noonan syndrome, caused by mutations in the PTPN11 gene and others, may also be associated with intestinal lymphangiectasia [10]. While genetic testing could identify potential causative mutations, such analysis is not routinely performed in clinical practice and was not performed in this case.

The mechanism of protein loss in intestinal lymphangiectasia is poorly understood, but increased pressure in the lymphatic vessels may be the cause [11]. In our case, lymphatic scintigraphy and MRL did not visualize lymphatic vessels downstream from the intestinal lymphatics. These findings may also suggest that this case lies toward the generalized end of the lymphatic anomaly spectrum, which may partly explain the observed response to sirolimus. Although imaging did not reveal any physical obstruction at the disconnected site, lymphatic flow may have been obstructed by hypoplasia, dysfunction, or stenosis of the lymphatic vessels, possibly increasing pressure within the vessels and leading to protein leakage. However, because the poorly imaged lymphatic vessels were not surgically removed for pathological evaluation, further cases are needed to confirm this hypothesis.

PIL treatments include nutritional therapy, medical management, surgery, and lymphatic embolization [4–6]. The mainstay treatment for PIL is nutritional therapy comprising a high‐protein, low‐fat diet, with restricted intake of long‐chain fatty acids and MCTs. This treatment decreases intestinal lymphatic flow and reduces intestinal loss [5, 6]. When nutritional therapy is ineffective, secondary treatments are recommended, including medical management, surgery, and lymphatic embolization [6]. Our patient was initially treated with a low‐strict fat diet with MCT supplementation and oral corticosteroids empirically to reduce intestinal permeability and possible inflammatory components, although the rationale for corticosteroid use in PIL remains limited and not well established. However, only partial improvement was achieved, indicating a need for secondary treatment. Considering the invasiveness of surgery and the limited availability of lymphatic embolization, drug therapy was selected.

The rationale for selecting sirolimus over other pharmacological options as secondary therapy in this case warrants discussion. Several medical treatments have been reported for PIL refractory to low‐strict fat diet with MCT supplementation, including octreotide, antiplasmin therapy (tranexamic acid), propranolol, and mTOR inhibitors (sirolimus and everolimus). Table 1 summarizes the reported pharmacological treatments for pediatric PIL. Octreotide, a somatostatin analog, is the most widely used pharmacological agent for PIL, with many cases showing clinical improvement, including increased serum albumin levels [4, 7, 12–14]. However, histological improvement varies, and treatment failure with fatal outcomes has been reported [4, 13, 14]. Tranexamic acid targets enhanced fibrinolytic activity implicated in intestinal protein loss, with reported clinical improvement in children [13] and symptom stabilization in combination with octreotide in refractory cases [7], though overall evidence remains limited. Propranolol affects lymphatic endothelium via VEGF suppression and capillary endothelial apoptosis, though treatment failure has been reported in pediatric PIL [11]. Among mTOR inhibitors, sirolimus suppresses mTOR signaling in lymphatic endothelial cells, inhibiting lymphatic vascular growth [4, 6, 15, 16], and has shown broad efficacy against vascular anomalies in a 2020 systematic review of 373 cases (lymphatic malformations: 94.9%) [17], though PIL cases may not have been included. Reports in PIL remain rare, especially in infants. In adults, sustained effects were achieved in 6 of 10 patients [18]; in pediatric cases, Kwon et al. [4] reported clinical improvement in all four children (100%) within 1–3 months.

**TABLE 1:** Reported pediatric pharmacologic treatments for primary intestinal lymphangiectasia (PIL).

Study (ref)	Age at diagnosis	Pharmacologic treatment	Time to response	Adverse events	Long‐term outcome/follow‐up
Sari et al. 2010 [12] (*n* = 6)	2–24 mo	Octreotide	NR	1 pancreatitis (long‐term use)	Decreased stool frequency and albumin infusion need; albumin normalized in 3/6; follow‐up: 15–64 mo
Kwon et al. 2021 [4] (adolescent case)	Adolescent	Octreotide ⟶ sirolimus	1 mo (sirolimus)	NR	Complete remission at 2 years; sirolimus maintained for 3 years
Kwon et al. 2021 [4] (neonatal case)	Neonate	Octreotide (failed) ⟶ sirolimus	1 mo (sirolimus)	NR	Paracentesis/thoracentesis no longer required; follow‐up: 4 mo
Kwon et al. 2021 [4] (2 additional cases)	Neonate/childhood	Sirolimus	Approximately 3 mo	NR	Gradual albumin increase; symptom improvement
MacLean et al. 2002 [13]	14 y	Octreotide ⟶ tranexamic acid	1 mo (tranexamic acid)	NR	Albumin increase; GI bleeding resolved; maintained stable with dose escalation
Prasad et al. 2019 [7] (*n* = 28)	1–18 y	Octreotide (*n* = 6), Tranexamic acid (*n* = 3)	NR	NR	Dietary responders maintained remission; relapse associated with poor compliance; median follow‐up: 39 mo
Ozeki et al. 2016 [11]	12 y	Propranolol (failed) ⟶ everolimus	4 weeks (everolimus)	NR	Diarrhea resolved; albumin gradually increased; no leakage at 6 mo; 12‐mo follow‐up
Acer‐Demir et al. 2020 [14]	3 y	Octreotide (refractory)	NR	NR	Required pancreas‐sparing duodenectomy; died 2 years later
Goret et al. 2025 [2] (*n* = 1)	NR	Octreotide (failed)	NR	NR	Complete remission under dietary therapy alone
Goret et al. 2025 [2] (*n* = 2)	NR	Sirolimus	NR	NR	Loss of efficacy at 18 mo (Case 2); inefficacy at 6 mo (Case 3); both discontinued
Goret et al. 2025 [2] (*n* = 1)	NR	Calciparin ⟶ octreotide	NR	NR	Transient improvement with calciparin for 5 years; octreotide ineffective; discontinued
Our case	7 mo	Sirolimus	1‐2 weeks	CK elevation (mild, reversible)	sustained remission; 17‐mo follow‐up

*Note:* “NR” indicates data not available in the original publications. mo = months; y = years. Time to response was measured from initiation of the specific agent noted in parentheses, not from the start of prior failed treatments.

As summarized in Table 1, pharmacologic therapies have demonstrated generally favorable response rates in reported pediatric cases, though complete biochemical normalization varies by agent [4, 12]. Regarding safety, although pancreatitis was reported in one case treated with octreotide [12], adverse events have not been reported in most pediatric PIL cases treated with these agents. Serious adverse events related to sirolimus have not been consistently reported in pediatric PIL [4], and in the present case, only mild and reversible CK elevation was observed. Sirolimus was selected in this patient based on its mechanism‐based targeting of lymphatic pathophysiology, its documented efficacy in refractory cases, and its established clinical experience in lymphatic malformations.

The treatment protocol for PIL with sirolimus has not been standardized. In this case, our approach was guided by clinical response and safety: (1) initial dosing of 0.5 mg/day (1.2 mg/m^2^/day) per Japanese package insert and consistent with previously reported doses of 1–1.6 mg/m^2^ [7]; (2) therapeutic drug monitoring (target 5–15 ng/mL) [3, 6]; (3) dose reduction for elevated creatine kinase, though causal relationship to sirolimus remains unclear; (4) continued treatment until clinical improvement and imaging resolution of protein leakage; and (5) discontinuation due to infection. Ideally, treatment duration and dose reduction should be individualized based on sustained clinical remission, normalized laboratory parameters, and imaging confirmation of disease resolution. Long‐term follow‐up for recurrence and nutritional status is essential. Recurrence should be managed individually based on treatment response and adverse events, potentially including sirolimus reinitiation or alternative therapies. Further case accumulation is needed to establish standardized management strategies for PIL. The reported time to response is 3‐4 weeks for octreotide, 4 weeks for sirolimus and everolimus, 2 weeks for propranolol, and 4 weeks for tranexamic acid [6]; early efficacy rates for sirolimus in refractory vascular anomalies have been reported as 35.7% at 7 days and 64.3% at 14 days [17]. In our case, a clinical response was observed within 2 weeks, with a sirolimus blood level of 7.2 ng/mL at that time, which was within the target range (5–15 ng/mL). This earlier‐than‐expected response may reflect the very early initiation of sirolimus (17 days after diagnosis), compared to previously reported intervals of 0.7–8.9 years [4]. These findings suggest that early introduction of sirolimus may contribute to a more rapid therapeutic effect in PIL. The patient experienced only mild, transient CK elevation with no other adverse events.

At 17 months of follow‐up, there was no recurrence of clinical symptoms, and nutritional status was favorable, as demonstrated by appropriate growth and development and laboratory findings. Notably, remission was maintained for 12 months after discontinuation of sirolimus. The optimal duration of mTOR inhibitor therapy and criteria for treatment discontinuation in lymphatic disorders remain unclear. This case suggests that sustained remission off therapy may be achievable in selected patients, although careful long‐term follow‐up is warranted.

Imaging evaluation helped determine the initiation of sirolimus treatment and understand the posttreatment state. We determined that secondary treatment was necessary based on laboratory parameters and persistent leakage on lymphatic scintigraphy and MRL. Furthermore, clinical improvement after sirolimus treatment was evaluated solely on laboratory parameters [4]; we demonstrated remission using posttreatment scintigraphy, confirming that protein leakage had ceased. Although no globally standardized criteria have been established, treatment response in PIL has been assessed using various parameters, including laboratory findings, imaging studies, and clinical symptoms [3]. We evaluated treatment efficacy using multiple objective parameters: normalization of serum protein levels, resolution of protein leakage on imaging studies, and clinical improvement of symptoms.

A limitation of this case is the concomitant use of corticosteroids at the time of sirolimus initiation, which limits attribution of the observed effects to sirolimus alone.

This case suggests one of the earliest reported responses to sirolimus in the treatment of PIL. Early introduction of sirolimus may be associated with rapid improvement in refractory PIL; however, this remains hypothesis‐generating, and further case accumulation is needed to confirm this association.

## Funding

No funding was received from any other sources for this case report.

## Ethics Statement

This study did not require Institutional Review Board approval, as it involves a single case report. All procedures performed in this case were conducted in accordance with the ethical standards of the Declaration of Helsinki.

## Consent

Written informed consent was obtained from the patient’s parents to publish this case report.

## Conflicts of Interest

The authors declare no conflicts of interest.

## Supporting Information

Additional supporting information can be found online in the Supporting Information section.

## Supporting information


**Supporting Information** CARE‐checklist‐English‐2013.

## Data Availability

The data that support the findings of this study are available on request from the corresponding author. The data are not publicly available due to privacy or ethical restrictions.
