# Supplementary material for: Successful Early Therapy With Sirolimus for an Infant With Primary Intestinal Lymphangiectasia
Source: Case Rep Pediatr. 2026 Sep 15;2026:7717714. doi: 10.1155/crpe/7717714 (PMC13579102; doi:10.1155/crpe/7717714)
Supplement: Supplementary file 1 [file CRPE-2026-7717714-s001.pdf]

CARE case report guidelines

CARE Checklist of information to include when writing a case report

(cc) BY-NC-ND

Topic	Item	Checklist item description	Reported on Line
**Title**	1	The diagnosis or intervention of primary focus followed by the words "case report"	1-2
**Key Words**	2	2 to 5 key words that identify diagnoses or interventions in this case report, including "case report"	47-48
**Abstract (no references)**	3a	Introduction: What is unique about this case and what does it add to the scientific literature?	20-25
	3b	Main symptoms and/or important clinical findings	25-33
	3c	The main diagnoses, therapeutic interventions, and outcomes	30-43
	3d	Conclusion—What is the main "take-away" lesson(s) from this case?	43-45
**Introduction**	4	One or two paragraphs summarizing why this case is unique (**may include references**)	50-89
**Patient Information**	5a	De-identified patient specific information.	90-96
	5b	Primary concerns and symptoms of the patient.	90-96
	5c	Medical, family, and psycho-social history including relevant genetic information	95-98
	5d	Relevant past interventions with outcomes	N/A
**Clinical Findings**	6	Describe significant physical examination (PE) and important clinical findings.	96-107
**Timeline**	7	Historical and current information from this episode of care organized as a timeline	108-151, Fig. 3
**Diagnostic Assessment**	8a	Diagnostic testing (such as PE, laboratory testing, imaging, surveys).	96-115, 127-145
	8b	Diagnostic challenges (such as access to testing, financial, or cultural)	108-109, 169-173
	8c	Diagnosis (including other diagnoses considered)	112-115, 166-182
	8d	Prognosis (such as staging in oncology) where applicable	N/A
**Therapeutic Intervention**	9a	Types of therapeutic intervention (such as pharmacologic, surgical, preventive, self-care)	116-143
	9b	Administration of therapeutic intervention (such as dosage, strength, duration)	116-143, 237-244
	9c	Changes in therapeutic intervention (with rationale)	129-143, 241-244
**Follow-up and Outcomes**	10a	Clinician and patient-assessed outcomes (if available)	137-151
	10b	Important follow-up diagnostic and other test results	143-151, 260-278
	10c	Intervention adherence and tolerability (How was this assessed?)	133-143, 245-259
	10d	Adverse and unanticipated events	141-151, 255-259
**Discussion**	11a	A scientific discussion of the strengths AND limitations associated with this case report	160-173, 279-282
	11b	Discussion of the relevant medical literature **with references**.	203-278
	11c	The scientific rationale for any conclusions (including assessment of possible causes)	222-278
	11d	The primary "take-away" lessons of this case report (without references) in a one paragraph conclusion	283-287
**Patient Perspective**	12	The patient should share their perspective in one to two paragraphs on the treatment(s) they received	N/A
**Informed Consent**	13	Did the patient give informed consent? Please provide if requested	Yes ☑ No ☐
